# Strategic Surgical Management of Idiopathic Iliac Vein Rupture: A Case Report Emphasizing Preoperative IVC Filter and Distal Venous Control

**DOI:** 10.3400/avd.cr.26-00011

**Published:** 2026-08-18

**Authors:** Keita Miyaishi, Moriyasu Nakaema, Masahiro Toyama, Shogo Niizaki, Shotaro Higa, Kojiro Furukawa

**Affiliations:** Department of Thoracic and Cardiovascular Surgery, Graduate School of Medicine, University of the Ryukyus, Ginowan, Okinawa, Japan

**Keywords:** idiopathic iliac vein rupture, deep vein thrombosis, phlegmasia cerulea dolens

## Abstract

Idiopathic iliac vein rupture (IIVR) is a rare, life-threatening condition. We report a successful case of a 66-year-old woman with hemorrhagic shock and disseminated intravascular coagulation managed with a strategic surgical approach. To prevent intraoperative pulmonary embolism from extensive deep vein thrombosis, a temporary inferior vena cava filter was placed. Distal venous control was achieved via a femoral vein clamp through a separate inguinal incision prior to pelvic exploration. Primary suture repair combined with thrombectomy successfully preserved venous outflow. This multistep strategy is effective for managing IIVR, ensuring hemodynamic stability and preventing fatal embolic complications.

## Introduction

Idiopathic iliac vein rupture (IIVR) is a rare clinical entity first described in 1961^1)^. By 2022, only approximately 70 cases had been reported worldwide.^1,2)^ The condition carries a high mortality rate, reported at approximately 20% overall, but potentially reaching 80% in patients presenting with hemodynamic collapse.^2,3)^

The underlying pathology often involves a combination of mechanical factors, such as iliac vein compression syndrome (May–Thurner syndrome), and acute factors like deep vein thrombosis (DVT).^4–6)^ However, preoperative diagnosis remains a significant challenge; nearly 85% of cases are only confirmed intraoperatively or via angiography.^2,4)^ Given the potential for misdiagnosis as an arterial rupture and the high risk of fatal pulmonary embolism during surgical manipulation, a standardized strategic approach is paramount.

## Case Report

A 66-year-old woman with a history of hypertension presented with sudden back pain and syncope. She was initially transported to another hospital, where contrast-enhanced computed tomography (CT) led to a diagnosis of a ruptured iliac artery. Upon referral to our institution, the patient was in clinical hemorrhagic shock with disseminated intravascular coagulation (DIC).

**Fig. 1:**
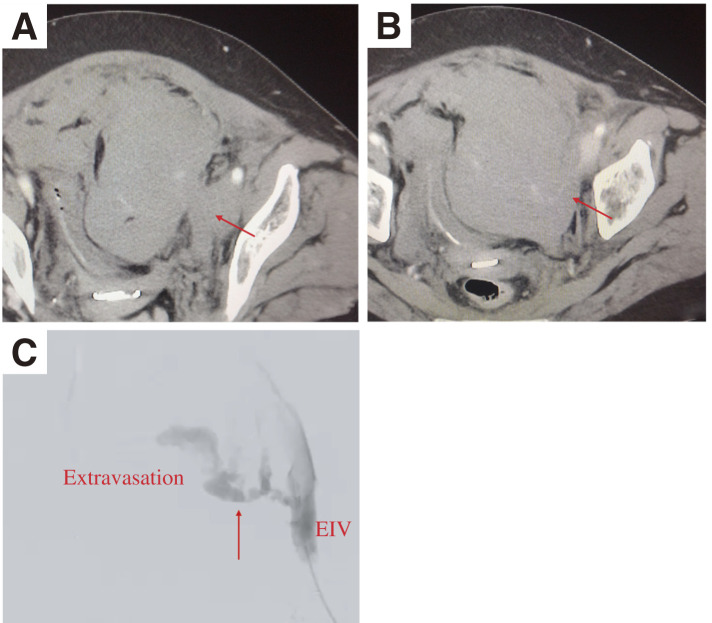
Preoperative and initial intraoperative diagnostic findings. (**A**) Preoperative contrast-enhanced CT showing the common iliac vein suspected to be obstructed (arrow). (**B**) CT image revealing extravasation continuous from the external iliac vein (arrow). (**C**) Initial intraoperative venography confirming extravasation from the external iliac vein (arrow) and the absence of the common and internal iliac veins due to obstruction. EIV, external iliac vein; CT, computed tomography

Physical examination revealed marked swelling and cyanosis of the left lower extremity. Re-evaluation of the CT images showed suspected venous extravasation and extensive thrombus occluding the entire length of the left common iliac vein. Because of the venous collapse and massive thrombosis, a definitive focal compression typical of May–Thurner syndrome could not be clearly identified at this stage. Nevertheless, these findings raised a strong suspicion of IIVR rather than an arterial event (**Fig. 1A** and **1B**).

The patient was immediately taken to the operating room. Because of the extensive DVT, we prioritized the prevention of pulmonary embolism by placing a temporary IVC filter (Günther Tulip; Cook Medical, Bloomington, IN, USA) via the contralateral healthy femoral vein at the start of the procedure.

The surgical approach was divided into 2 stages to ensure controlled hemostasis. First, a longitudinal incision was made in the left inguinal region. We carefully identified the femoral vein; notably, the great saphenous vein was dilated to 20 mm, requiring caution to avoid misidentification. The femoral vein was secured to provide distal control. A 4-Fr sheath was inserted through this secured vein, and intraoperative angiography confirmed the rupture site in the external iliac vein and complete occlusion of the common and internal iliac veins (**Fig. 1C**).

**Fig. 2:**
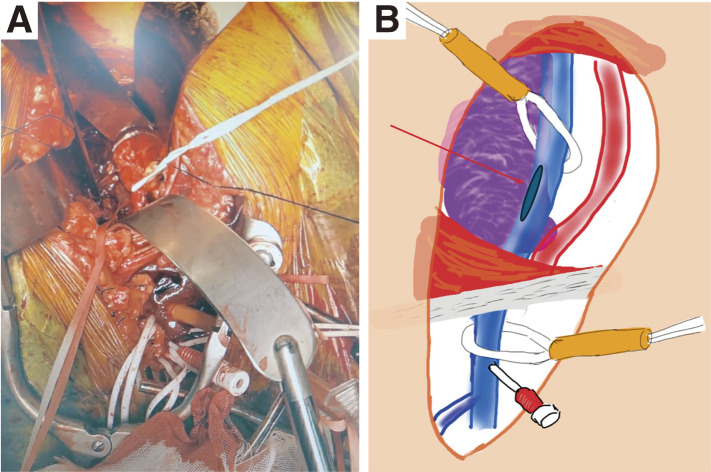
Surgical strategy and intraoperative completion. (**A**) Intraoperative photograph. (**B**) Intraoperative schema illustrating the pararectus incision with preservation of the inguinal ligament. The hematoma is removed, the external iliac vein is secured, and distal control is achieved by clamping the femoral vein via a separate inguinal incision. The arrow indicates the bleeding site.

Next, the incision was extended cephalad to a pararectus line, approaching the retroperitoneal space while preserving the inguinal ligament. Upon evacuating a large hematoma, venous hemorrhage was encountered in the pelvis. A tourniquet was applied to the previously secured femoral vein to control the bleeding. Because the clamped external iliac vein collapsed, making the exact tear difficult to find, we gently injected saline through the pre-placed femoral sheath. This maneuver distended the vein without wasting blood, allowing us to accurately pinpoint a 3-cm longitudinal rupture at the midpoint of the external iliac vein (**Fig. 2**). Macroscopically, the ruptured vein wall appeared normal, without obvious fragility or any remarkable changes.

**Fig. 3:**
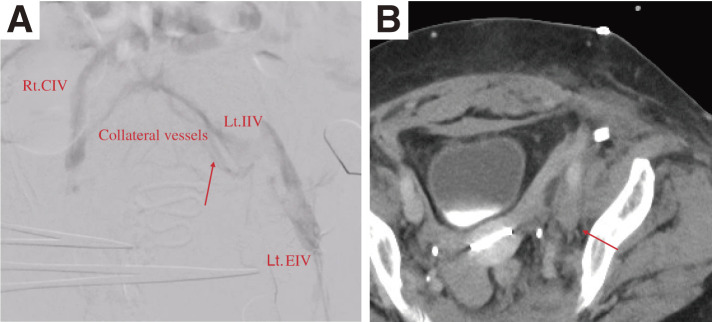
Postoperative outcomes. (**A**) Final intraoperative venography showing a patent venous flow from the external iliac vein to the contralateral iliac vein via collateral vessels and the internal iliac vein. (**B**) Postoperative contrast-enhanced CT in the delayed phase showing the patent external iliac vein and established collateral vessels leading to the anterior sacral surface (arrow), along with the resolution of the retroperitoneal hematoma without signs of further extravasation. CIV, common iliac vein; EIV, external iliac vein; IIV, internal iliac vein; Lt, left; Rt, right; CT, computed tomography

Thrombectomy was performed using a 4-Fr Fogarty balloon. During withdrawal from the common iliac vein, the balloon encountered significant resistance and was deformed, suggesting an underlying stenosis. After thrombectomy, central backflow was confirmed. Rather than ligation, we performed a primary suture repair to maintain venous drainage and prevent worsening of lower extremity stasis. Final angiography confirmed that while the common iliac vein remained occluded, the external iliac vein was patent, with blood draining through the internal iliac vein and anterior sacral venous plexus to the contralateral side (**Fig. 3A**). The total operative time was 236 min, and the estimated blood loss was 1535 mL, including the evacuated retroperitoneal hematoma.

The postoperative course was stable. The patient’s critical limb congestion (phlegmasia) had resolved rapidly via collateral drainage, successfully preserving the limb. Unfractionated heparin was initiated on postoperative day 2 via continuous intravenous infusion. The dosage was carefully titrated from 5000 U/day to 10000 U/day under strict monitoring of bleeding tendencies, including drain output and coagulation profiles (e.g., hemoglobin, fibrinogen, and activated partial thromboplastin time). Oral anticoagulation (edoxaban, 30 mg/day) was initiated on postoperative day 9, following the resolution of DIC. Postoperative contrast-enhanced CT performed 1 week later demonstrated the patency of the repaired external iliac vein and the establishment of collateral vessels leading to the anterior sacral surface. Furthermore, the massive retroperitoneal hematoma had successfully been resolved without any signs of further extravasation (**Fig. 3B**). The CT also revealed the affected iliac vein diameter to be approximately 16 mm. While typical mechanical compression by the right common iliac artery, characteristic of May–Thurner syndrome, was not evident, a moderate stenosis of undetermined etiology was observed at the origin of the left common iliac vein.

The IVC filter was removed on postoperative day 9, and the patient was discharged in good condition on day 34. While moderate residual edema persisted, we managed it conservatively with continuous anticoagulation and compression therapy, without delayed stenting. Over a 1-year follow-up period, the residual edema resolved.

## Discussion

This case highlights 3 critical strategic pitfalls in the management of IIVR.

### 1. The diagnostic challenge

Preoperative diagnosis is notoriously difficult, with correct CT-based diagnosis reported in only about 10% of cases.^2)^ The low-pressure nature of the venous system means that extravasation may not be as prominent as in arterial ruptures. Clinicians should maintain a high index of suspicion for IIVR in patients with non-traumatic pelvic hemorrhage, especially when accompanied by signs of phlegmasia cerulea dolens or when DVT is present. Delayed-phase CT imaging is essential to identify venous extravasation. Furthermore, when preoperative imaging is inconclusive, a distally placed femoral sheath allows for intraoperative venography, providing definitive anatomical mapping of the rupture site. Beyond formal venography, this diagnostic sheath serves a secondary critical role during open exploration: once the vein is clamped for hemostasis and consequently collapses, gentle saline injection through the sheath enables hydrodistention. This simple yet highly effective maneuver visually unmasks the precise tear without inducing further obscuring hemorrhage.

### 2. Surgical control in venous bleeding

The accumulation of a massive pelvic hematoma can obscure the bleeding point, making vessel taping in the pelvis nearly impossible immediately after hematoma removal. Our strategy of securing the femoral vein in the inguinal region before entering the retroperitoneal space provided reliable distal control. Furthermore, the initial inguinal incision can simply be extended cephalad into a pararectus line, eliminating the need for a separate abdominal incision. This continuous incision provides sufficient retroperitoneal exposure while keeping the inguinal ligament intact.

Although initial central control may seem less critical due to the underlying proximal venous occlusion, successful thrombectomy that restores essential venous drainage will inevitably produce central back-bleeding. To manage this expected and necessary backflow, the same Fogarty balloon catheter used for thrombectomy can simply be kept inflated proximally, serving as an effective intraluminal occluder.

### 3. Maintaining venous outflow vs. hemostasis

To contextualize our strategy, a brief review of the literature is warranted. According to a recent systematic review by Li et al., which analyzed 68 reported cases of IIVR, the overall mortality remains significant. In their review, the mortality rate for open surgical repair was reported at 17.65%, whereas all 12 patients who received endovascular treatment (EVT) survived. However, as explicitly demonstrated in their multivariate analysis, open surgery itself did not increase the risk of death. They noted that the higher observed mortality associated with open repair is primarily because these procedures are often performed as emergency laparotomies without an accurate preoperative diagnosis. Furthermore, they acknowledged that critical variables, such as the true degree of shock and the amount of bleeding in these severe cases, were frequently unrecorded in previous reports.^2)^

Historically, open surgery with venous ligation was the standard approach for rapid hemostasis, although it is associated with severe chronic venous insufficiency and increased morbidity.^2)^ Alternatively, EVT utilizing covered stents has emerged as a less invasive approach to achieve hemostasis and reduce venous hypertension,^7)^ and bare-metal stents are considered to recanalize underlying venous stenosis.^6,8)^

In our case, while acute extensive thrombosis obscured classical CT findings of May–Thurner syndrome, the combination of intraoperative Fogarty resistance and the delayed identification of a proximal venous stenosis strongly suggests that an underlying structural stricture was the fundamental predisposing factor. Although this theoretically made stenting an ideal approach to address the underlying stenosis and maintain venous outflow, EVT has notable limitations in highly acute and unstable settings. Deploying any stent in the presence of profound hemorrhagic shock, DIC, and extensive DVT carries a highly elevated risk of early stent occlusion, proximal thrombus extension, and fatal pulmonary embolism.^9,10)^ Furthermore, EVT does not allow for the direct evacuation of a massive retroperitoneal hematoma. Beyond these general limitations, specifically in our case, the affected iliac vein was completely obscured by the massive hematoma and thrombosis on the acute-phase CT, making accurate sizing and safe stent deployment clinically unfeasible. Consequently, EVT was deferred in favor of definitive open surgery.

Even in the subacute phase, delayed stenting was not deemed feasible. Our experience demonstrates that, at the time, the lack of appropriately sized venous stents made repurposing arterial stents unacceptably risky due to potential migration or inadequate expansion. However, achieving patency up to the external and internal iliac veins alone fostered sufficient collateral drainage, allowing for the complete, albeit gradual, resolution of edema over approximately 1 year.

This clinical course provides reassuring insight that aggressive recanalization of the common iliac vein is not strictly mandatory when the procedure proves clinically prohibitive. Nevertheless, the recent emergence of venous-dedicated stents suggests a promising alternative. In the current era, these devices might enable early and safe anatomical recanalization in similar cases, potentially accelerating the resolution of residual edema and improving early quality of life.

Because the management of IIVR requires conflicting goals of surgical hemostasis and early anticoagulation for DVT, ensuring surgical stability through a definitive open repair is a prerequisite for initiating safe anticoagulation.

Finally, the risk of fatal pulmonary embolism during thrombectomy or vein repair must not be overlooked. The placement of a temporary IVC filter is a vital adjunct in cases where DVT is identified preoperatively.

## Conclusion

Successful management of IIVR depends on early recognition, the use of preoperative IVC filters to prevent embolism, and a systematic surgical approach that includes early distal control and the preservation of venous outflow through suture repair.
